# Coronary artery lesions in children with Kawasaki disease: status quo and nursing care

**DOI:** 10.3389/fcvm.2024.1272475

**Published:** 2024-04-22

**Authors:** Cen Chen, Qiuhui Chen, Tong Zhang, Yanping Ling

**Affiliations:** Department of Nursing, Children’s Hospital of Nanjing Medical University, Nanjing, China

**Keywords:** coronary artery lesions, children, Kawasaki disease, care, treatment

## Abstract

**Aim:**

Coronary artery lesion (CAL) is a common yet serious complication in children with Kawasaki disease. The aim of the present study was to evaluate the influencing factors of CAL in children with Kawasaki disease, to provide reference for the clinical treatment and care of children with Kawasaki disease.

**Design:**

A retrospective cohort study.

**Methods:**

Children with Kawasaki disease treated in a tertiary hospital in China between 1 January 2021 and 31 December 2022 were selected. The characteristics and clinical data of children with Kawasaki disease were analyzed. Spearman’s correlation analysis was conducted to evaluate the relationship between CAL and the characteristics of children with Kawasaki disease. A logistic regression analysis was used to analyze the influencing factors of CAL in children with Kawasaki disease.

**Results:**

In total, 185 children with Kawasaki disease were included; the incidence of CAL in children with Kawasaki disease was 18.38%. Pearson’s correlation analysis showed that gender (*r* = 0.504), age (*r* = 0.611), duration of fever ≥10 days (*r* = 0.579), hemoglobin (Hb) (*r* = 0.623), and C-reactive protein (CRP) (*r* = 0.558) were all correlated with the CAL in children with Kawasaki disease (all *p* < 0.05). Logistic regression analyses showed that male [odds ratio (OR) = 2.543, 95% confidence interval (CI): 1.801–3.077, *p* = 0.040], age ≤2 years (OR = 3.002, 95% CI: 2.744–3.641, *p* = 0.012), duration of fever ≥10 days (OR = 2.089, 95% CI: 1.624–2.515, *p* = 0.028), Hb ≤105 g/L (OR = 1.914, 95% CI: 1.431–2.406, *p* = 0.013), and CRP ≥100 mg/L (OR = 2.168, 95% CI: 1.893–2.531, *p* = 0.035) were the risk factors of CAL in children with Kawasaki disease (all *p* < 0.05).

**Conclusions:**

The incidence of CAL in children with Kawasaki disease is high and there are many related risk factors. Clinical medical workers should take early warning and carry out interventions and nursing care according to these risk factors to improve the prognosis of children with Kawasaki disease.

## Background

Kawasaki disease is a systemic vasculitis syndrome with unknown etiology, which usually occurs in children aged under 5 years (1). It can involve all the organs in the body, in addition to the skin, lymph nodes, and heart, but also affect the kidney, lung, liver, spleen, and other organs. Coronary artery lesions (CAL) are the most serious (2, 3). In recent years, due to increased understanding of Kawasaki disease and attention to early diagnosis and treatment, the case fatality rate of Kawasaki disease in the acute and subacute phases has dropped to less than 0.1% (4). It has been reported that the incidence of Kawasaki disease is increasing year by year, and has replaced rheumatic fever as the main cause of acquired heart disease in children (5, 6). How to predict the occurrence of CAL in advance and intervene as soon as possible to reduce the occurrence and development of coronary artery disease has become an urgent problem for clinical medical workers.

At present, the pathogenesis of Kawasaki disease is still not completely clear. The clinical diagnosis of Kawasaki disease is still based on the typical symptoms of children. Through clinical practice, it has been found that it is easy for Kawasaki disease to involve systemic blood vessels and damage coronary arteries (7). CAL, as a common and serious complication of Kawasaki disease, may induce ischemic heart disease in the later stage, and even more serious myocardial infarction, threatening the physical and mental health and quality of life of children (8–10). Therefore, the early detection and diagnosis of Kawasaki disease and scientific and effective early intervention measures have an important impact on improving the prognosis of children. Therefore, the aim of the present study was to analyze the current situation and related risk factors of CAL in children with Kawasaki disease, in order to provide a reference basis for clinical treatment and nursing care of Kawasaki disease.

## Methods

### Ethics

This study used a retrospective cohort design. The study protocol had been checked and approved by the ethical committee of Children's Hospital of Nanjing Medical University (approval number: 201701010). All the children's information was processed anonymously, and the collected data were used only for this study.

### Study population

In this study, children with Kawasaki disease treated in a Chinese tertiary hospital between 1 January 2021 and 31 December 2022 were selected as the study population. The inclusion criteria of the children in this study were as follows: age less than 13 years; diagnosed with Kawasaki disease for the first time and received treatment in our hospital; and the clinical data of the child were complete. We excluded children with other systemic diseases and incomplete clinical data.

### Diagnostic criteria

The diagnostic criteria (11, 12) of Kawasaki disease were that the child had a fever for ≥5 days and at least four of the following main symptoms: (1) bilateral bulbar conjunctival congestion; (2) non-suppurative swelling of cervical lymph nodes in the acute stage; (3) diffuse hyperemia of lip redness, chapped, myrica tongue and pharynx; (4) pleomorphic rash; and (5) acute palmoplantar erythema, hard edema of hands and feet, membrane-like exfoliation at the migration of the nail bed of the skin in the convalescent stage. Four of the above main clinical manifestations appeared, but echocardiography showed CAL, but not other diseases, and could also be diagnosed as Kawasaki disease.

Two investigators (CC and QC) assessed the CALs in this study. The diagnostic criteria of CAL in this study were as follows: using Cardio *Z* software; the absolute value of the coronary artery examined by echocardiography was converted into a *Z* value, and the *Z* value internal diameter of any segment of coronary artery ≥2.0. Typically, in Kawasaki disease, CALs are defined as having a *Z*-score of 2.5 or higher, as commonly reported in the literature. The *Z*-score assessments used in this report are derived from Chinese reports (12–15).

### Treatments

After diagnosis, children with Kawasaki disease were given gamma globulin (Sichuan Yuanda Shuyang Pharmaceutical Co., Ltd., national medicine standard: S10980026, specification: 2.5 g/branch) 2 g kgT'-dMel 1 by intravenous drip. At the same time, 30–50 mg kg'-d' enteric-coated aspirin (Bayer Medical and Health Co., Ltd., national medicine standard: H20171021, specification: 0.1 g/tablet) was given orally three times. After 72 h of fever, the dosage of aspirin was adjusted to 5 mg kg'-dT' three times daily for 12 weeks. Patients with coronary artery injury stopped using aspirin after the coronary artery returned to normal. The patients among them with elevated platelet and coronary artery injury were treated with aspirin.

### Data collection

We collected and analyzed the clinical data of children with Kawasaki disease. The relevant characteristics and data collected in this study included gender, age, body mass index (BMI), duration of fever, clinical symptoms including changes of oral mucosa, enlarged lymph nodes, skin rash, and conjunctival congestion. We also collected the following laboratory indicators: hemoglobin (Hb), white blood cell count (WBC), C-reactive protein (CRP), platelet count (PLT), erythrocyte sedimentation rate (ESR), aspartate aminotransferase (AST), and alanine aminotransferase (ALT). The patients were assessed with blood tests and CALs at the time of admission and once a week after admission. All the data were collected by the two authors from the medical records system using a unified form for collection, and the data were verified and compared in the later stage to ensure their authenticity.

### Statistical method

In this study, SPSS version 23.0 statistical software was used for the data analysis. The chi-square test was used to compare the classification variables represented by numbers and proportions. All the data were examined for normal distributions. We carried out univariate and multivariate logistic regression analyses to screen the predictors. Spearman’s correlation analysis was conducted to evaluate the relationship between CAL and the characteristics of children with Kawasaki disease. A logistic regression analysis was used to analyze the influencing factors of CAL in children with Kawasaki disease. In this study, *p* < 0.05 showed that the difference between groups was statistically significant.

## Results

### Characteristics of children

In total, 185 children with Kawasaki disease were included; 34 children had been diagnosed with CAL, giving an incidence of 18.38%. As indicated in Table 1, there were significant differences in the gender, age, duration of fever ≥10 days, enlarged lymph nodes, and skin rash between children with and without CAL (all *p* < 0.05). There were no significant differences in the BMI, changes of oral mucosa, and conjunctival congestion between the children with and without CAL (all *p* > 0.05).

**Table 1:** The characteristics of children with Kawasaki disease (*n* = 185).

Factors	CAL group (*n* = 34)	No CAL group (*n* = 151)	*t*/*χ*^2^	*p*
Male/female	24/10	82/69	1.264	0.007
Age (years)	1.14 ± 0.99	2.85 ± 1.41	1.118	0.016
BMI (kg/m^2^)	19.47 ± 2.09	20.01 ± 2.55	3.034	0.089
Duration of fever ≥10 days	9 (26.47%)	7 (4.64%)	1.227	0.001
Changes of oral mucosa	26 (76.47%)	109 (72.19%)	2.085	0.103
Enlarged lymph nodes	20 (58.82%)	114 (75.50%)	1.712	0.049
Skin rash	16 (47.06%)	102 (67.55%)	1.438	0.024
Conjunctival congestion	27 (79.41%)	116 (76.82%)	2.255	0.092

BMI, body mass index; CAL, coronary artery lesion.

As indicated in Table 2, there were significant differences in Hb and CRP between children with and without CAL (all *p* < 0.05). There were no significant differences in WBC, PLT, ESR, AST, and ALT between the children with and without CAL (all *p* > 0.05).

**Table 2:** The laboratory test results children with Kawasaki disease.

Variables	CAL group (*n* = 34)	No CAL group (*n* = 151)	*t*	*p*
Hb (g/L)	92.26 ± 10.44	116.03 ± 13.71	12.715	0.013
WBC (×10^9^/L)	14.72 ± 5.06	14.29 ± 6.63	4.964	0.083
CRP (mg/L)	116.13 ± 20.33	79.09 ± 15.73	8.375	0.001
PLT (×10^9^/L)	364.19 ± 110.21	353.25 ± 104.48	22.536	0.118
ESR (mm/h)	36.75 ± 12.61	33.17 ± 14.05	3.087	0.073
AST (U/L)	69.02 ± 20.14	65.22 ± 17.60	11.054	0.107
ALT (U/L)	74.32 ± 21.85	76.96 ± 20.18	12.199	0.094

CAL, coronary artery lesion; Hb, hemoglobin; WBC, white blood cell count; CRP, C-reactive protein; PLT, platelet count, ESR, erythrocyte sedimentation rate; AST, aspartate aminotransferase; ALT, alanine aminotransferase.

### Relationship of CAL and characteristics of children with Kawasaki disease

As indicated in Table 3, Spearman’s correlation analysis showed that gender (*r* = 0.504, *p* = 0.012), age (*r* = 0.611, *p* = 0.009), duration of fever ≥10 days (*r* = 0.579, *p* = 0.005), Hb (*r* = 0.623, *p* = 0.031), and CRP (*r* = 0.558, *p* = 0.018) were associated with the CAL in children with Kawasaki disease.

**Table 3:** Spearman correlation analysis on the relationship of CAL and characteristics of children with Kawasaki disease.

Characteristic	*r*	*p*
Gender	0.504	0.012
Age (years)	0.611	0.009
BMI (kg/m^2^)	0.034	0.089
Duration of fever ≥10 days	0.579	0.005
Changes of oral mucosa	0.104	0.236
Enlarged lymph nodes	0.127	0.099
Skin rash	0.114	0.066
Conjunctival congestion	0.096	0.124
Hb (g/L)	0.623	0.031
WBC (×10^9^/L)	0.107	0.068
CRP (mg/L)	0.558	0.018
PLT (×10^9^/L)	0.113	0.154
ESR (mm/h)	0.219	0.098
AST (U/L)	0.095	0.114
ALT (U/L)	0.105	0.083

BMI, body mass index; CAL, coronary artery lesion; Hb, hemoglobin; WBC, white blood cell count; CRP, C-reactive protein; PLT, platelet count, ESR, erythrocyte sedimentation rate; AST, aspartate aminotransferase; ALT, alanine aminotransferase.

### Risk factors of CAL in children with Kawasaki disease

Table 4 shows the variable assignment of multivariate logistic regression in this study. As indicated in Table 5, the logistic regression analyses showed that being male [odds ratio (OR) = 2.543, 95% confidence interval (CI): 1.801–3.077, *p* = 0.040], age ≤2 years (OR = 3.002, 95% CI: 2.744–3.641, *p* = 0.012), duration of fever ≥10 days (OR = 2.089, 95% CI: 1.624–2.515, *p* = 0.028), Hb ≤105 g/L (OR = 1.914, 95% CI: 1.431–2.406, *p* = 0.013), and CRP ≥100 mg/L (OR = 2.168, 95% CI: 1.893–2.531, *p* = 0.035) were the risk factors of CAL in children with Kawasaki disease (all *p* < 0.05).

**Table 4:** The variable assignment of multivariate logistic regression.

Factors	Variables	Assignment
CAL	Y	Yes = 1, no = 2
Gender	X_1_	Male = 1, female = 2
Age (years)	X_2_	≤2 = 1, >2 = 2
Duration of fever ≥10 days	X_3_	Yes = 1, no = 2
Hb (g/L)	X_4_	≤105.5 = 1, >105.5 = 2
CRP (mg/L)	X_5_	≥100 = 1, <100 = 2

CAL, coronary artery lesion; Hb, hemoglobin; CRP, C-reactive protein.

**Table 5:** The logistic regression analysis on the risk factors of CAL in children with Kawasaki disease.

Variables	*β*	S · $\bar{x}$	OR	95% CI	*p*
Male	0.182	0.112	2.543	1.801–3.077	0.040
Age ≤2 years	0.177	0.129	3.002	2.744–3.641	0.012
Duration of fever ≥10 days	0.205	0.151	2.089	1.624–2.515	0.028
Hb ≤105 g/L	0.216	0.118	1.914	1.431–2.406	0.013
CRP ≥100 mg/L	0.199	0.104	2.168	1.893–2.531	0.035

CAL, coronary artery lesion; Hb, hemoglobin; CRP, C-reactive protein.

## Discussion

Kawasaki disease is a systemic vasculitis disease with unclear etiology. CAL is the main complication of Kawasaki disease, and a small number of children may have coronary artery stenosis or thrombus, or even sudden death, which is the main factor affecting the long-term prognosis of Kawasaki disease (16, 17). A multicenter epidemiological survey (18) showed that the incidence of Kawasaki disease complicated with CAL has gradually increased in recent years. Timely diagnosis and treatment to reduce the incidence of CAL is still the main problem in the treatment and nursing care of Kawasaki disease. It is reported that up to 25.9% of children with Kawasaki disease have coronary artery disease and 1.8% of patients with Kawasaki disease have coronary artery aneurysms (19). To further find and understand the clinical characteristics and related risk factors of Kawasaki disease complicated with CAL is the focus of early diagnosis, which can provide necessary theoretical support for the treatment and nursing for these children. The results of this retrospective cohort have found that the incidence of CAL in children with Kawasaki disease is 18.38%, and being male, aged ≤2 years, a duration of fever ≥10 days, Hb ≤105 g/L, and CRP ≥100 mg/L are the risk factors of CAL in children with Kawasaki disease. Clinical medical workers should be alerted to the possibility of CAL in these children with risk factors, and screen, diagnose, and begin nursing care as early as possible to reduce the occurrence and development of CAL.

Being male is a high-risk factor for Kawasaki disease complicated with CAL. An epidemiological investigation in Jilin Province shows that the incidence of male children is 1.83 times higher than that of female children (20). At present, the incidence of Kawasaki disease in male children is higher than that in female children in most studies, but there is no significant difference in coronary artery disease between the male and female children in some reports (21–23). Haijian et al. (24) found that the male-to-female ratio of children with Kawasaki disease aged less than 6 months can reach 9:1. Previous studies (25–27) found that 88.9% of children with Kawasaki disease are under the age of 5 years old. The results of an epidemiological investigation in China (28) showed that CAL are most common in children aged under 1 year, and the age of coronary artery disease is 8.4 months. Some studies (29–31) have found that the clinical manifestations of children aged under 1 year, especially those aged under 6 months, are very atypical; some symptoms and signs appear late or do not appear, and the misdiagnosis rate is high. Therefore, the actual incidence of Kawasaki disease in infants may be higher than that reported. In addition to the simple analysis of age and gender, an analysis combining age and gender may point to the next direction in research, which needs further investigation.

At present, Kawasaki disease lacks specific diagnostic indicators and clinicopathological features. CRP is a kind of cellular acute phase reaction, which increases in the acute phase and decreases in the recovery stage (32). It changes dynamically during the course of Kawasaki disease. Its elevated level reflects the degree of inflammation of the body, and the severity of inflammatory reaction is positively correlated with CRP level (33). Many studies (34–36) have shown that CRP can aggravate vascular inflammatory injury through cytokines, and the increase of CRP is a high-risk factor for CAL. Therefore, the timely detection of Hb and CRP levels is of great significance to evaluate CALs in children with Kawasaki disease.

The treatment of children with Kawasaki disease in the acute stage is mainly anti-platelet aggregation and reducing systemic non-specific vascular inflammation to reduce and avoid the occurrence of coronary artery injury (37, 38). It has been reported that once the child has been diagnosed and a high-dose intravenous drip of Immunoglobulin combined with aspirin treatment, the prognosis of the child was good; all of them had improved or been cured (39, 40). Because of the high incidence and harmfulness of Kawasaki disease, clinicians should be more vigilant against Kawasaki disease so that those cases with unknown causes of fever and oral mucosal changes should be highly suspected to be Kawasaki disease (41, 42). For those who do not meet the diagnostic criteria, we should refer to the laboratory test results to improve the diagnostic accuracy of Kawasaki disease, and it is necessary to fully grasp the high-risk factors of Kawasaki disease. This will be of great significance in reducing cardiovascular complications associated with Kawasaki disease.

The present study has some limitations. First, the sample size of this study is small; the limited sample size may be underpowered to detect the related factors. Second, as this study is a retrospective cohort, there is the possibility of various biases. Further conclusions need to be confirmed by more rigorous, multicenter, large-sample prospective studies. Clinically, healthcare providers should pay attention to high-risk children with timely evaluation, diagnosis, and early treatment, and strengthen the long-term management and follow-up of children with CAL to improve the prognosis of children with Kawasaki disease.

## Conclusion

In summary, this study has found that the incidence of CAL in children with Kawasaki disease is 18.38%, and being male, aged ≤2 years, duration of fever ≥10 days, Hb ≤105 g/L, and CRP ≥100 mg/L are the risk factors of CAL in children with Kawasaki disease.

## Data Availability

The original contributions presented in the study are included in the article/Supplementary Material, further inquiries can be directed to the corresponding author.
